# cDNA Cloning, Bioinformatics, and Expression Analysis of *ApsANS* in *Acer pseudosieboldianum*

**DOI:** 10.3390/ijms26051865

**Published:** 2025-02-21

**Authors:** Mingrui Li, Zhuo Weng, Zihan Gong, Xiaoyu Li, Jiayi Ye, Yufu Gao, Liping Rong

**Affiliations:** College of Agriculture, Yanbian University, Yanji 133000, China

**Keywords:** ANS, anthocyanins, cDNA clone, bioinformatics, expression analysis, *Acer pseudosieboldianum* (Pax) Komarov

## Abstract

Anthocyanin synthetase (ANS), a key enzyme in the final step of the anthocyanin synthesis pathway, catalyzes the conversion of leucoanthocyanidins to anthocyanins. In this study, an ANS structural protein (TRINITY_DN18024_c0_g1) was found to be associated with anthocyanin accumulation in *Acer pseudosieboldianum* leaves, named *ApsANS*. Real-time quantitative fluorescence PCR analysis revealed that the expression of *ApsANS* was significantly higher in red-leaved (variant) than green-leaved (wild-type) strains, which was consistent with the transcriptome data. The UPLC results showed that the cyanidin metabolites may be the key substance influencing the final color formation of *Acer pseudosieboldianum*. The *ApsANS* gene was cloned and analyzed through bioinformatics analysis. *ApsANS* has a total length of 1371 bp, and it encodes 360 amino acids. Analysis of the structural domain of the *ApsANS* protein revealed that *ApsANS* contains a PcbC functional domain. Protein secondary structure predictions indicate that α-helix, irregularly coiled, and extended chains are the major building blocks. Subcellular localization predicted that *ApsANS* might be localized in the nucleus. The phylogenetic tree revealed that *ApsANS* is relatively closely related to *ApANS* in *Acer palmatum*. The prediction of miRNA showed that the *ApsANS* gene is regulated by miR6200. This study provides a theoretical reference for further analyzing the regulatory mechanism of leaf color formation in *Acer pseudosieboldianum*.

## 1. Introduction

Anthocyanidins are important products in the flavonoid metabolic pathway that determine the color of most angiosperm tissues, such as flowers, leaves, fruits, and episperm [1,2]. Anthocyanins are natural water-soluble plant pigments that not only enhance the color of plants but also show many bioactive properties, such as anti-inflammatory, antioxidant, and anticancer activities [3,4]. The precursor of anthocyanin biosynthesis is phenylalanine, which is catalyzed by a series of enzymes to produce anthocyanins [5], and the key enzymes involved in this process include phenylalanine ammonia lyase (PAL), cinnamate-4-hydroxylase (C4H), chalcone synthase (CHS), chalcone isomerase (CHI), flavanone-3-hydroxylase (F3H), dihydroflavonol-4-reductase (DFR), and anthocyanidin synthase (ANS) [6,7,8]. ANS is one of the important downstream enzymes, and it catalyzes the conversion of leucoanthocyanidins into colored anthocyanins. It was first isolated from a *Zea mays* mutant by using transposon tagging and subsequently isolated from *Arabidopsis thaliana* [9,10], *Malus pumila* [11], *Vitis vinifera* [12], *Litchi chinensis* [13], *Mangifera indica* [14], *Camellia sinensis* [15], *Hosta plantaginea* (Lam.) [16], *Dioscorea opposite,* and *Punica granatum* L. [17,18]. It has been shown that overexpression of the anthocyanin synthase gene *StANS* can promote anthocyanin accumulation in potato tubers [19]. It was reported that the anthocyanin and chlorophyll contents in *Arabidopsis* lines with the lowest ANS expression levels were lower [20]. It has been found that the expression of ANS iso-structural genes correlates well with fruit coloration and anthocyanin accumulation [21]. Previous studies on ANS have focused more on the flower or fruit color of horticultural plants but less on woody foliage plants; consequently, the characteristics and expression of ANS in foliage plants remain unclear [22,23].

Ornamental plants are an important part of agriculture and horticulture [24]. Among them, leaf color is one of the most important ornamental features [25]. Some studies have shown that the change in leaf color of colorful ornamental plants is related to the synthesis and accumulation of anthocyanin in the leaves [26,27]. *Acer* refers to the genus *Acer* in the family *Aceraceae*, and, as an important ornamental tree species, it is found in Asia, Europe, North America, and the northern edge of Africa [28,29,30]. China is the main distribution area of *Acer* species, with more than 150 species of germplasm resources, accounting for more than half of the world’s *Acer* resources [31]. *Acer* species are mostly arbors, which are widely used in the timber industry due to their tall, straight trunks [32]. *Acer* also has medicinal value, and the extracts from different tissues of the *Acer* tree contain a wealth of nutrients, such as amino acids, fatty acids, and minerals, and some key physiological active substances, such as triterpenes, chlorogenic acid, and neuronic acid [33]. In addition, many *Acer* species exhibit different types of leaf color changes during growth and development, which play an important role in enriching the color of native forests [34]. *Acer pseudosieboldianum* is a genus of *Acer* in the family Aceraceae, which includes mostly deciduous trees or shrubs that are mainly distributed in eastern Heilongjiang, eastern Liaoning, and southeastern Jilin [35]. Its tree posture and leaf shape, and especially its red leaves in autumn, are of great ornamental value; therefore, it is an important landscape tree species. Research on *Acer pseudosieboldianum* has focused on resource evaluation, physiological characteristics, tissue culture, and environmental factors; however, few studies have been conducted on the mechanism of leaf coloration, which has seriously impeded the progress of research on molecular breeding for relevant leaf color [36]. In our previous study, we found a mutant with green and red leaves in spring and summer, respectively. Transcriptome sequencing revealed that the differential gene ANS is one of the key factors that affects variations in leaf color [37].

Therefore, in the present study, we obtained the candidate ANS gene by screening the transcriptome database, analyzed the expression level of the ANS gene in variant and wild-type strains through real-time PCR (RT-PCR), analyzed its structural characteristics and physicochemical properties, and predicted its binding to miRNAs through bioinformatics analysis. The present study aimed to establish the molecular foundation for further understanding *ApsANS* gene regulation of leaf color and to provide a theoretical reference for leaf color formation mechanisms, expression regulation, and ornamental enhancement of *Acer pseudosieboldianum*.

## 2. Results

### 2.1. Screening and Expression Analysis of the ANS Gene in Acer pseudosieboldianum

**Figure 1:**
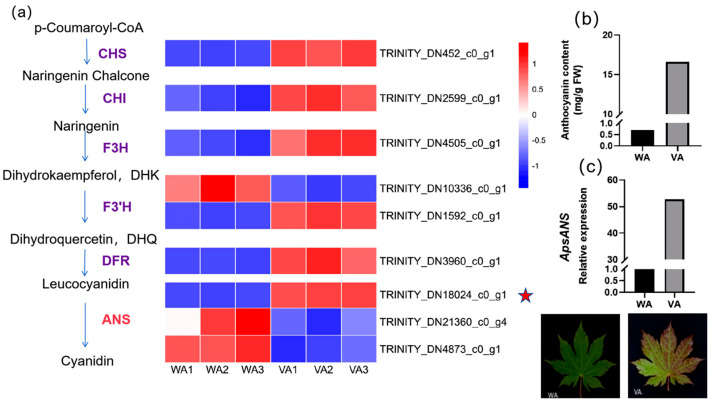
Determination of anthocyanin content and gene expression analysis. (**a**) Expression profiles of structural genes in wild-type *Acer pseudosieboldianum* and variants. The heatmap on the right is based on the RPKM values of the corresponding transcripts in the transcriptome database. (**b**) Comparison of anthocyanin content between wild type and variant of *Acer pseudosieboldianum*. ‘WA’ represents the wild-type strain, and ‘VA’ represents the variant strain. (**c**) Relative expression of *ApsANS* in wild type and variants.

In this study, we screened the transcriptome to identify nine structural genes that were differentially expressed in the wild-type (WA) and variant (VA) leaves of *Acer pseudosieboldianum* during anthocyanin biosynthesis, and we plotted heatmaps based on their FPKM values, in which the expression of ANS, a key enzyme at the end of the anthocyanin biosynthesis pathway, plays an important role in the coloration of *Acer pseudosieboldianum* leaves. Among the three *ANS* genes screened, only the *ApsANS* (TRINITY_DN18024_c0_g1) gene had very low expression in the green leaves (WA) and high expression in the red leaves (VA); the expression was consistent with leaf color (Figure 1a). Afterwards, the relative expression of the *ApsANS* gene in the leaves of wild-type and variant strains of *Acer pseudosieboldianum* was detected through real-time fluorescence quantitative PCR (Figure 1c), and the expression of the *ApsANS* gene was significantly lower in the wild strain than in the variegated strain. The anthocyanin content in the mature leaves of *Acer pseudosieboldianum* was determined spectrophotometrically, and the total anthocyanin content detected in the variegated strain was 16.63 mg/g, while the wild type of *Acer pseudosieboldianum* contained only a small amount of anthocyanin (0.7 mg/g), with the anthocyanin content of the variegated strain being about 23.76 times higher than that of the wild type (Figure 1b), and the trend of anthocyanin content was consistent with the trend of *ApsANS* gene expression, suggesting that the *ApsANS* gene may be a key gene for leaf pigmentation in *Acer pseudosieboldianum*. 

### 2.2. Screening and Quantitative Analysis of Differential Metabolites

**Figure 2:**
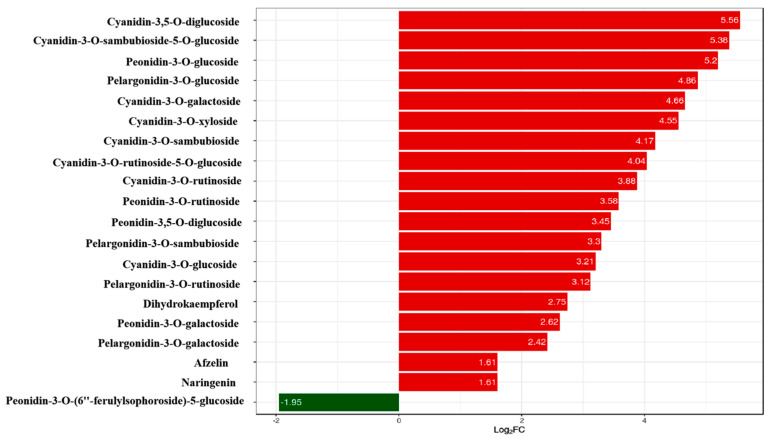
Differential metabolites in wild type and variants. The horizontal coordinate is the log_2_FC of the differential metabolites; vertical coordinates are differentially expressed metabolites. The red color represents up-regulation of differentially expressed metabolites, and the green color represents down-regulation of differentially expressed metabolites.

**Figure 3:**
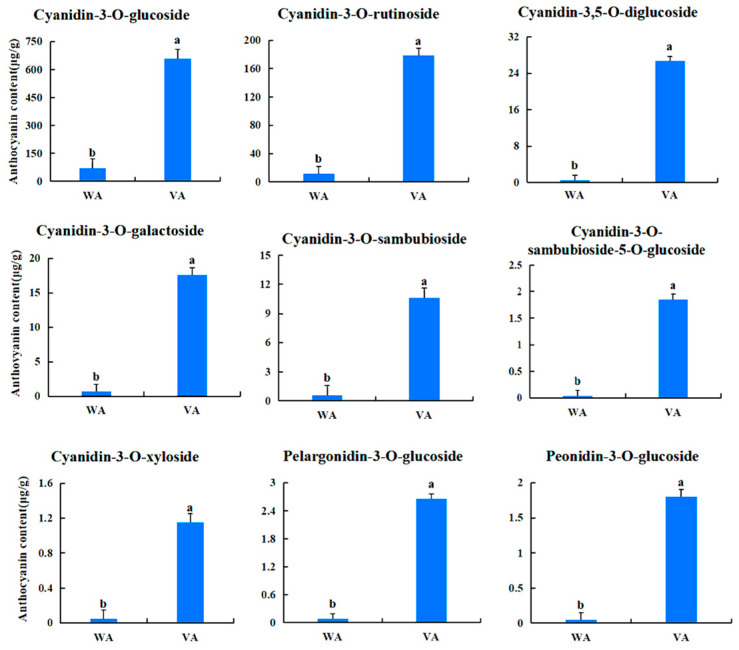
Content of each anthocyanoside metabolite in wild-type and variant *Acer pseudosieboldianum*. Different letters indicate significant differences (*p* < 0.05).

In order investigate the pigment composition affecting the coloration of *Acer pseudosieboldianum* leaves, we screened and quantified differential metabolites in wild-type and variant *Acer pseudosieboldianum* leaves through UPLC. We selected metabolites with a multiplicity of difference greater than 1.5 to plot a bar graph, as shown in Figure 2, which contained eight cyanidin types (cyanidin-3,5-O-diglucoside, cyanidin-3-O-sambubioside-5-O-glucoside, cyanidin-3-O-galactoside, cyanidin-3-O-xyloside, cyanidin-3-O-sambubioside, cyanidin-3-O-rutinoside-5-O-glucoside, cyanidin-3-O-rutinoside, cyanidin-3-O-glucoside), five peonidin types (peonidin-3-O-glucoside, peonidin-3-O-rutinoside, peonidin-3,5-O-diglucoside, peonidin-3-O-galactoside, peonidin-3-O-(6″-ferulylsophoroside)-5-glucoside), four pelargonidin types (pelargonidin-3-O-glucoside, pelargonidin-3-O-sambubioside, pelargonidin-3-O-rutinoside, pelargonidin-3-O-galactoside) and three flavonoid metabolites (dihydrokaempferol, afzelin, naringenin). Among these, cyanidin-3,5-O-diglucoside showed the largest difference between the wild type and the variant. After that, we quantitatively analyzed the differential metabolites (Figure 3), and the results showed that the cyanidin metabolites were the highest in the leaves of *Acer pseudosieboldianum*, and the content of the wild type was significantly lower than that of the variant strain. In particular, cyanidin-3-O-glucoside was the highest in the leaves of *Acer pseudosieboldianum*, which indicated that it might be one of the important pigments affecting the coloration of the leaves of *Acer pseudosieboldianum*.

### 2.3. Cloning and Characterization of ApsANS cDNA

**Figure 4:**
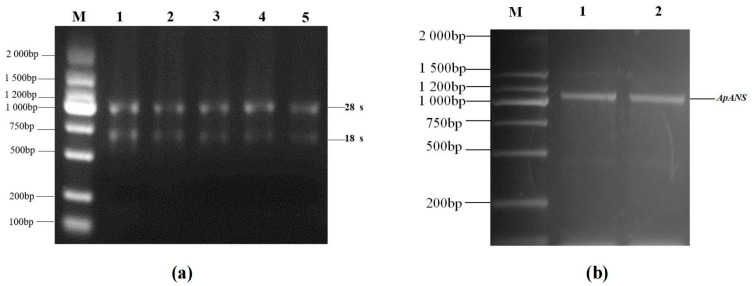
cDNA amplification of the *ApsANS* gene in *Acer pseudosieboldianum*. (**a**) Lane 1–5: total RNA. (**b**) Lane 1–2: PCR product band based on *Acer pseudosieboldianum* cDNA template. (Lane M: DL2000 DNA marker).

The RNA was extracted from *Acer pseudosieboldianum* leaves using the TIANGEN RNA Easy Fast Plant Tissue kit. The 28S, 18S, and 5S bands were clearly visible (Figure 4a), which were used for gene cloning. The TIANGEN Fast King cDNA First-Strand Synthesis kit was used for reverse transcription of the mRNA into cDNA. On the basis of the *ApsANS* fragment sequence obtained from the *Acer pseudosieboldianum* genomic data, a conserved fragment primer was designed for PCR amplification, and gel electrophoresis showed a clear bright band at approximately 1000 bp (Figure 4b).

### 2.4. Physicochemical Properties of the ApsANS Protein

The basic physicochemical properties of the *ApsANS* protein were analyzed through ExPASy. The results are shown in Table 1. The *ApsANS* gene has a full length of 1371 bp and a maximum open reading frame (ORF) of 1083 bp; it encodes a 360-amino-acid protein with a molecular weight of 40.684 kDa, a theoretical isoelectric point of 5.84, an instability coefficient of 49.75, an average hydrophilicity of −0.413, and a lipid coefficient of 89.58.

**Figure 5:**
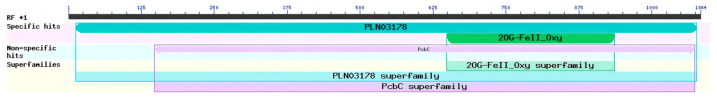
Prediction of conserved domain of the *ApsANS* protein.

**Figure 6:**
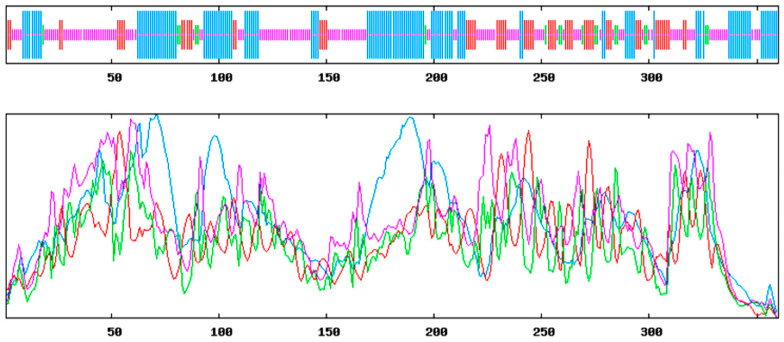
Secondary structure prediction of *ApsANS*. Alpha helix (blue); Random coil (purple); Extended strand (red); Beta turn (green).

The prediction of the conserved structural domains of the *ApsANS* protein using the CD-search function on the NCBI website revealed that it contains the pcbC functional domain (Figure 5), with a conserved α-ketoglutarate and a divalent iron ion-dependent double oxygenase superfamily domain [2OG-Fe(II)_Oxy] at position 650–935 of the peptide chain. The results of secondary and tertiary structure prediction of the *ApsANS* protein showed that the structures mainly consisted of α-helix, irregular coils, and extended chains, which accounted for 35.56%, 17.50%, and 41.94% of the total structure, respectively (Figure 6).

### 2.5. Structural Analysis of the ApsANS Protein

**Figure 7:**
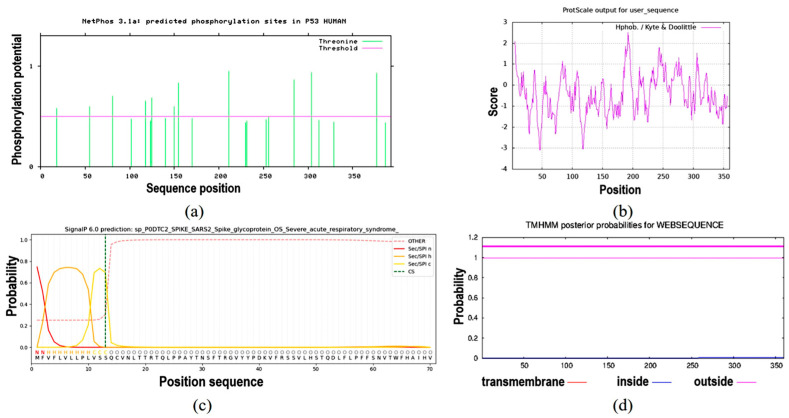
Prediction of hydrophobicity, transmembrane helix, signal peptide, and phosphorylation of the *ApsANS* protein. (**a**) Phosphorylation site prediction. (**b**) Prediction of hydrophobicity or hydrophilicity. (**c**) Signal peptides and their site prediction on proteins. (**d**) Prediction of transmembrane structural domains.

**Figure 8:**
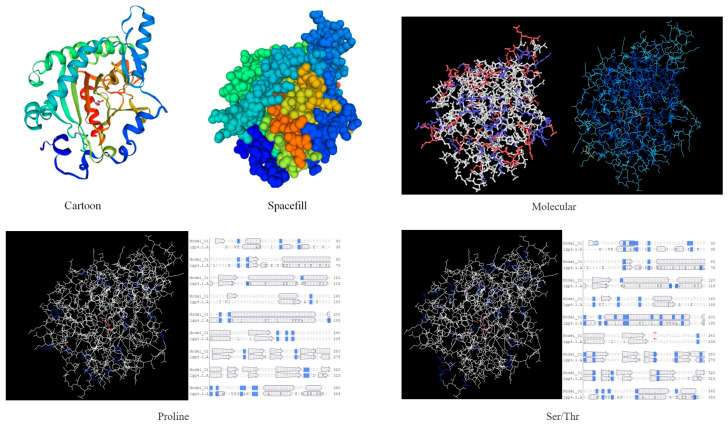
3D structure prediction of the *ApsANS* protein.

NetPhos 3.1 prediction analysis revealed that the protein contained 22 Thr phosphorylation sites (Figure 7a). The ProtScale online tool was used to predict the hydrophobic domain of the *ApsANS* protein from *Acer pseudosieboldianum*. The results showed that the *ApsANS* protein has one hydrophobic domain in the regions 20–36, 186–195, and 235–249 of its amino acid sequence (Figure 7b), which contains a mitochondrial targeting peptide, no chloroplast transport peptide, and a secretory pathway signaling peptide (Figure 7c). TMHMM predictions show that the protein does not have a transmembrane helix (Figure 7d). Subcellular localization prediction showed that the *ApsANS* protein was localized in the cytoplasm. The 3D structure of the *ApsANS* protein was further predicted using SWISS-MODEL. The *ApsANS* protein belongs to the α-ketoglutarate dioxygenase family, and the spatial structure is dominated by α-helix and random coiling; these findings are consistent with the predicted results of the secondary structure (Figure 8).

### 2.6. miRNA Prediction of ApsANS in Acer pseudosieboldianum

The miRNA prediction of *ApsANS* was performed using the psRNATarget database (Table 2). The prediction results showed that the *ApsANS* gene might be regulated by miR6200, with the following sequence: UUUGGCCAACUAGAUCUAUGA.

### 2.7. Multiple Sequence Alignment and Evolutionary Tree Construction for the ApsANS Protein

**Figure 9:**
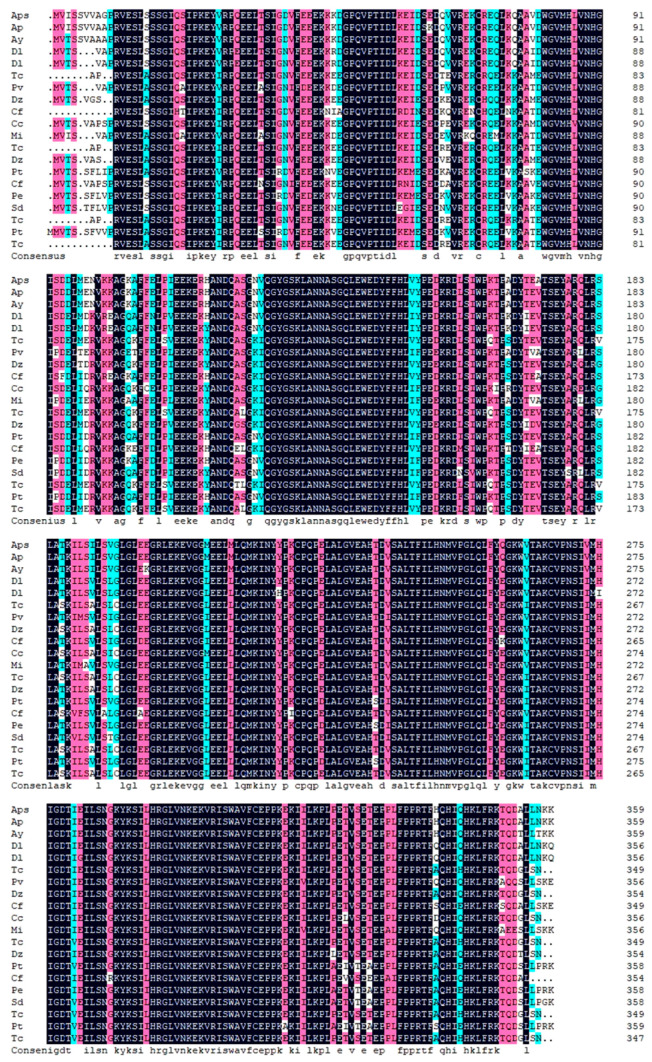
Multiple sequence alignment of the predicted amino acid sequence of *ApsANS* with other ANS proteins. (The species names corresponding to the abbreviated sections can be found in Abbreviations).

Multiple sequence comparison and phylogenetic tree construction of *Acer pseudosieboldianum ApsANS* with ANS protein sequences from other species are shown in Table A1. The amino acid sequences of species with high homology to the *ApsANS* protein were downloaded through the BLAST program in the NCBI database. Multiple comparisons of the homologous sequences were performed using DNAMAN software version 9.0, and the sequences of *Acer palmatum* (AWN08246.1), *Dimocarpus longan* (QRV61372.1), *Dimocarpus longan* (ACK76231.1), *Pistacia vera* (XM_031428804.1), and *Mangifera indica* (XM_044630098.1) were observed to be highly homologous to the *ApsANS* protein (Figure 9).

**Figure 10:**
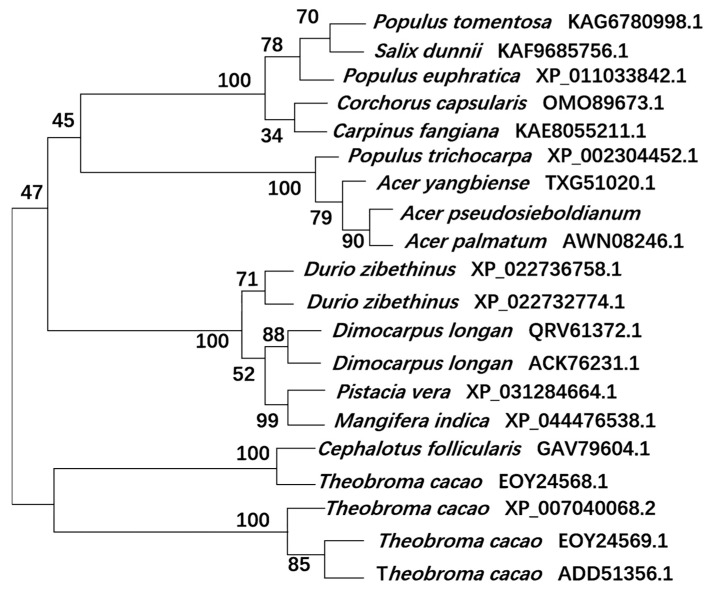
Evolutionary analysis of the *ApsANS* system.

To further understand the evolutionary relationships between *ApsANS* and other plant ANS proteins, the NJ method using MEGA7.0 software was used to construct a phylogenetic tree, and all of the species were divided into four major taxa (Figure 10). The evolutionary relationship between *ApsANS* from *Acer pseudosieboldianum*, *Acer palmatum*, *Acer yangbiense*, and *Populus trichocarpa*, the family and genus rank of *ApsANS*, are distinguished from each other, and the evolution is basically in accordance with the plant’s taxonomic classification. *Acer pseudosieboldianum* and *Ace palmatum* share the closest evolutionary relationship. This may be because both *Acer pseudosieboldianum* and *Ace palmatum* are maple plants, and the ANS may have the same functions; hence, they share close evolutionary relationships. In general, the plant’s evolutionary relationships remained largely consistent with the results of the amino acid comparison.

## 3. Discussion

Anthocyanins and other flavonoids are the main pigments that determine the coloration of many plant tissues and organs [1]. Color changes in a variety of plants have been associated with anthocyanin biosynthesis, such as those reported for lichee (*Litchi chinensis* Sonn) [13], mango (*Mangifera indica* Linn) [14], strawberry (*Fragaria* × *ananassa*) [38], alfalfa (*Medicago sativa*) [39], white clover (*Trifolium repens*) [40], white primrose (*Primula vulgaris*) [41], and other plants. Anthocyanin biosynthesis is one of the most deeply studied plant secondary metabolic pathways, and its synthesis is highly conserved [42]. In most plant species, anthocyanins are generated through a branch of the flavonoid biosynthetic pathway with a group of catalytic enzymes [43,44]. Anthocyanin synthase (ANS) is a key enzyme gene expressed at the final step of the anthocyanin biosynthesis pathway, which belongs to the dioxygenase gene family in the flavonoid pathway and relies on the α-ketoglutarate ion and the Fe^2+^ ion to catalyze the conversion of colorless anthocyanins into colored anthocyanins [45,46,47], which plays an important role in regulating flower color, leaf color formation, and fruit coloration [48,49,50].

In this study, we analyzed the expression pattern of the *ApsANS* gene in the mature leaves of *Acer pseudosieboldianum* wild-type and variant strains, and we found that the expression of the *ApsANS* gene was higher in the red variant leaf and lower in the green leaf, which was consistent with the transcriptome data and the anthocyanin content (Figure 1), suggesting that *ApsANS* may be related to the pigmentation of *Acer pseudosieboldianum* leaves. The UPLC results showed that the cyanidin metabolites may be the key substance influencing the final color formation of *Acer pseudosieboldianum* (Figure 2 and Figure 3). The *ApsANS* gene was successfully isolated from the leaves of *Acer pseudosieboldianum* through PCR (Figure 4). Analysis of the structural domains of the *ApsANS* protein revealed that the protein contains a pcbC functional domain and a conserved α-ketoglutarate and divalent iron ion-dependent dioxygenase superfamily region [2OG-Fe (II)_Oxy] at position 650–935 in the peptide chain (Figure 5 and Figure 6). The predicted secondary and tertiary structures of the *ApsANS* protein showed that the structures mainly included α-helix, irregular coils, and extended chains, which accounted for 35.56%, 17.50%, and 41.94% of the total structures, respectively (Figure 6). The *ApsANS* protein has a hydrophobic structural domain that contains a mitochondrial targeting peptide, no chloroplast transit peptide, and no secretory pathway signaling peptide, but the protein has no transmembrane structural domains. The 3D structure prediction revealed that the spatial structure of the *ApsANS* protein is dominated by α-helices and random coiling and that it belongs to the α-ketoglutarate dioxygenase family, and these findings are consistent with the predictions of the secondary structure (Figure 7 and Figure 8). Subcellular localization predicted that the *ApsANS* protein might be localized in the nucleus. BLAST comparison and phylogenetic tree construction showed that the *ApsANS* protein is relatively closely related to the *ApANS* protein (Figure 9 and Figure 10). miRNA molecules bind to the target miRNA through base pairing, which causes translation inhibition of the target miRNA after transcription, thus affecting the expression of protein [51,52]. In plants, miRNA can participate in gene expression during the synthesis of flavonoids, thus affecting the accumulation of flavonoids in leaves [53,54,55]. The present study predicted that miR6200 may regulate the expression of the *ApsANS* gene and affect the synthesis of flavonoids (Table 2). Specific mechanisms of action need to be further investigated. In the next step, we will construct an overexpression vector for the *ApsANS* gene, and because the genetic transformation of *Acer pseudosieboldianum* is relatively difficult, we will choose to carry out stable transformation in Nicotiana benthamiana and analyze the role of MYB, bHLH, and WD40 regulatory factors on the *ApsANS* gene in order to further verify the function and clarify the molecular regulation mechanism of the *ApsANS* gene in the leaf color formation of *Acer pseudosieboldianum*. Our research findings are expected to provide a theoretical basis for future trait improvement of *Acer pseudosieboldianum*.

## 4. Materials and Methods

### 4.1. Plant Material, Strains, and Vectors

*Acer pseudosieboldianum* was used as the test material and grown in the nursery of Yanbian University in Jilin Province, China. Three-year old *Acer pseudosieboldianum* variant leaves were collected for ANS gene cloning. The red leaf variant was instantly frozen in liquid nitrogen and stored at −80 °C for gene cloning. Mature leaves of the variant (VA) and wild-type (WA) strains were also collected for leaf color expression analysis. The RNA extraction kit, high-fidelity enzymes and other reagents, and the reverse transcription kit were purchased from TIANGEN (Beijing, China). Escherichia coli DH5-α strains were obtained from TaKaRa (Dalian, China), and primer synthesis and sequencing were performed by Sangon Biotech (Shanghai, China).

### 4.2. Isolation of Total RNA and Synthesis of First-Strand cDNA

RNA was extracted from the leaves of *Acer pseudosieboldianum* using the RNA Easy Fast Plant Tissue kit (TIANGEN, Beijing, China), and the quality was checked using 1.0% agarose gel electrophoresis. After its concentration was measured using a spectrophotometer and its integrity was validated through gel electrophoresis, the first-strand cDNA was synthesized through reverse transcription with the total RNA as the template in accordance with the instructions of the Fast King cDNA First-Strand Synthesis kit (TIANGEN, Beijing, China). The cDNA synthesized through reverse transcription was used as the template, and Primer 5.0 software was used to design specific primers as follows: ApsANS-F: ATGGTGATTTCATCGGTAGTAGCA; ApsANS-R: TTAGACTTTTTTGTTG AGGAGAGAGCA. The corresponding upstream and downstream primers were used for PCR amplification to clone *ApsANS*. The PCR was performed as follows: initial denaturation at 94 °C for 5 min, 32 cycles of amplification, each consisting of a denaturing step at 94 °C for 30 s, followed by annealing at 57 °C for 30 s and extension at 72 °C for 2 min. The last cycle was followed by a 5 min extension at 72 °C; subsequently, the PCR products were stored at 4 °C and examined through gel electrophoresis. The target gene fragments with the correct band size were recovered and purified using the agarose gel DNA recovery kit (TIANGEN, Beijing, China). In accordance with the instructions of the pMD19-T vector kit (TaKaRa, Dalian, China), the gel recovery product was ligated overnight with the pMD19-T vector. The ligation product was transformed into E. coli DH5α (TaKaRa, Dalian, China) receptor cells, after which the cells were shaken uniformly in LB solid medium containing ampicillin and incubated at 37 °C until white single colonies appeared on the medium. PCR amplification was performed to detect the presence or absence of target bands. The PCR reaction procedure was as follows: initial denaturation at 95 °C for 10 min, 32 cycles of amplification, each consisting of a denaturing step at 95 °C for 15 s, followed by annealing at 56 °C for 30 s and extension at 72 °C for 2 min. The last cycle was followed by a 5 min extension at 72 °C.

### 4.3. Determination of Anthocyanin Content

The anthocyanin extraction of *Acer pseudosieboldianum* followed the previous method, with some modifications [56,57]. We weighed 0.1 g of leaf samples in a 50 mL centrifuge tube, added 1% HCl–methanol solution to 20 mL, shook it gently and mixed it well, and then immersed it in 25 °C for 5–6 h, shaking every 2 h. The filtrate was filtered, and the supernatant was collected after centrifugation. The standard buffer was used as a blank control, and the absorbance values were detected at 530 nm and 600 nm with a ultraviolet spectrophotometer (INESA, Shanghai, China). Three biological replicates were performed, and the anthocyanin content was calculated as D_530nm_ − D_600nm_ = 0.01 (U).

### 4.4. Quantification of Anthocyanin Metabolites Through UPLC-ESI-MS/MS

Quantification of Anthocyanin Metabolites were detected by MetWare (Hubei, China) based on the AB Sciex QTRAP 6500 LC-MS/MS platform. The sample was freeze-dried, ground into powder (30 Hz, 1.5 min), and stored at −80 °C until needed. Then, 50 mg of powder was weighted and extracted with 0.5 mL of methanol/water/hydrochloric acid (500:500:1, *v*/*v*/*v*). Then, the extract was vortexed for 5 min, followed by ultrasound for 5 min, and then it was centrifuged at 12,000× *g* under 4 °C for 3 min. The residue was re-extracted by repeating the above steps again under the same conditions. The supernatants were collected and filtrated through a membrane filter (0.22 μm, Anpel, Shanghai, China) before LC-MS/MS analysis. The sample extracts were analyzed using an UPLC-ESI-MS/MS system (UPLC, ExionLC™ AD, SCIEX, The Netherlands; MS, Applied Biosystems 6500 Triple Quadrupole, SCIEX, The Netherlands). The analytical conditions were as follows. UPLC: column, WatersACQUITY BEH C18 (1.7 µm, 2.1 mm × 100 mm); solvent system, water (0.1% formic acid):methanol (0.1% formic acid); gradient program, 95:5 *v*/*v* at 0min, 50:50 *v*/*v* at 6 min, 5:95 *v*/*v* at 12 min, hold for 2 min, 95:5 *v*/*v* at 14 min; hold for 2 min; flow rate, 0.35 mL/min; temperature, 40 °C; injection volume, 2 µL. Quantification of metabolites was performed using scheduled multiple reaction monitoring (MRM) through mass spectrometry.

### 4.5. Screening and Expression Analysis of ANS Genes

Heat mapping of related structural genes was conducted using the Hiplot online tool (https://hiplot.com.cn/cloud-tool, accessed on 27 November 2024). To verify the correctness of the expression pattern of the candidate genes, qRT-PCR was performed to validate the *ApsANS* gene, which is associated with anthocyanin synthesis. cDNAs from wild-type and variant leaves of *Acer pseudosieboldianum* were used as templates for quantitative analysis using a fluorescence quantification kit (SYBR^®^ Premix Ex Taq, TaKaRa, Dalian, China) and a Gene9600 real-time fluorescence quantitative PCR instrument (BIOER, Hangzhou, China) with the following primer sequences: ApsANS-F: GAAAAGGAAGTAGGTGGTATGG; ApsANS-R: CAATGGTGTCTCCGATGTGC. The reaction procedure was 95 °C for 3 min, 95 °C for 10 s, annealing temperature of 58 °C for 30 s, and 39 cycles; finally, the lysis curve program was added. The relative gene expression of each sample was calculated using the 2^−△△Ct^ method [58].

### 4.6. Analysis of Physicochemical Properties of the ApsANS Protein

The ProtParam tool in the ExPASY server (https://web.expasy.org/protparam/, accessed on 22 March 2022) was used to predict the molecular weight, isoelectric point, instability coefficient, lipid coefficient, and hydrophobicity of *ApsANS* [59]. Online software SOPMA 2.0 (https://npsa-prabi.ibcp.fr/cgi-bin/npsa_automat.pl?Page=npsa_sopma.html, accessed on 24 March 2022) was used to predict the secondary structure of the protein.

### 4.7. Conservative Domain of the ApsANS Protein and miRNA Prediction

By using MEME program V5.3.2 (https://meme-suite.org/meme/tools/meme, accessed on 23 March 2022), the structure of the *ApsANS* protein was predicted under the assumption that it is a conserved sequence, with the following parameters: number of repetitions, nonspecific; width of the motif, 6–50; number of motifs, 6; other parameters, default values. All motifs recognized by the MEME program were searched using the InterProScan program (http://www.ebi.ac.uk/interpro/search/sequence/, accessed on 23 March 2022) in the InterPro database [60]. The online tool psRNATarget (https://www.zhaolab.org/psRNATarget/, accessed on 7 April 2022) was used for target prediction of miRNA [53].

### 4.8. Sequence Alignment Analysis and Construction of Phylogenetic Tree

Mega7.0 software was used to construct the system evolution tree and for sequence analysis. The amino acid sequences of the *ApsANS* protein of *Acer pseudosieboldianum* were compared and analyzed. The comparison results were used to construct the phylogenetic tree through the neighbor joining (NJ) method, with 1000 bootstrap replicates and default values for other parameters [61].

## 5. Conclusions

In this study, the expression pattern, structural characteristics, and physicochemical properties of the anthocyanin synthase gene *ApsANS* in *Acer pseudosieboldianum* were analyzed. Gene expression analysis showed that the expression of the *ApsANS* gene in the variant was significantly higher than in the wild type, and the trend was consistent with the anthocyanin content. The cyanidin metabolites may be key substances in the formation of *Acer pseudosieboldianum* colors. The *ApsANS* gene belongs to the α-ketoglutarate dioxygenase family, and it has a pcbC functional domain, which may be regulated by miR6200 to affect flavonoid synthesis. This study further explored the expression pattern of the *ApsANS* gene in *Acer pseudosieboldianum* and provided some theoretical basis for breeding *Acer pseudosieboldianum* varieties rich in anthocyanins.

## Figures and Tables

**Table 1:** Physicochemical properties of the *ApsANS* protein.

Amino Acid Number	Molecular Weight (kDa)	pI	Instability	Hydropathicity
360	40.684	5.84	49.75	−0.413

**Table 2:** *ApsANS* miRNA prediction.

Target Gene	miRNA Expectation	Target Accessibility	Target Beginning and End	miRNA Sequence
*ApsANS*	miR6200	4	474~494	UUUGGCCAACUAGAUCUAUGA

## Data Availability

The sequenced raw reads generated in this study have been submitted to the National Center for Biotechnology Information (NCBI), with BioProject ID PRJNA805289.

## Abbreviations

Aps	*Acer pseudosieboldianum*
Ap	*Acer palmatum*
Ay	*Acer yangbiense*
Dl	*Dimocarpus longan*
Tc	*Theobroma cacao*
Pv	*Pistacia vera*
Dz	*Durio zibethinus*
Cf	*Cephalotus follicularis*
Cc	*Corchorus capsularis*
Mi	*Mangifera indica*
Pt	*Populus tomentosa*
Cf	*Carpinus fangiana*
Pe	*Populus euphratica*
Sd	*Salix dunnii*
Pt	*Populus trichocarpa*

## Appendix A

**Table A1:** The ANS protein sequence for constructing the phylogenetic tree.

Species Names	GenBank ID
*Acer palmatum*	AWN08246.1
*Acer yangbiense*	TXG51020.1
*Dimocarpus longan*	QRV61372.1
*Dimocarpus longan*	ACK76231.1
*Theobroma cacao*	XP_007040068.2
*Pistacia vera*	XP_031284664.1
*Durio zibethinus*	XP_022736758.1
*Cephalotus follicularis*	GAV79604.1
*Corchorus capsularis*	OMO89673.1
*Mangifera indica*	XP_044476538.1
*Theobroma cacao*	EOY24569.1
*Durio zibethinus*	XP_022732774.1
*Populus tomentosa*	KAG6780998.1
*Carpinus fangiana*	KAE8055211.1
*Populus euphratica*	XP_011033842.1
*Salix dunnii*	KAF9685756.1
*Theobroma cacao*	ADD51356.1
*Populus trichocarpa*	XP_002304452.1
*Theobroma cacao*	EOY24568.1
